# Implementation of Wearable Technology for Remote Heart Rate Variability Biofeedback in Cardiac Rehabilitation

**DOI:** 10.3390/s25030690

**Published:** 2025-01-24

**Authors:** Tiehan Hu, Xianbin Zhang, Richard C. Millham, Lin Xu, Wanqing Wu

**Affiliations:** 1 School of Biomedical Engineering, Sun Yat-Sen University, Shenzhen 518107, China; huth3@mail2.sysu.edu.cn (T.H.); zhangxy785@mail2.sysu.edu.cn (X.Z.); 2 Department of Information Technology, Durban University of Technology, Durban 4001, South Africa; richardm1@dut.ac.za; 3 General Hospital of the Southern Theatre Command, Guangzhou 510010, China; 4 The First School of Clinical Medicine, Southern Medical University, Guangzhou 510515, China

**Keywords:** cardiac rehabilitation, wearable technology, human-machine collaboration, personalized biofeedback

## Abstract

Cardiovascular diseases pose a significant threat to global health, and cardiac rehabilitation (CR) has become a critical component of patient care. Heart Rate Variability Biofeedback (HRVB) is a non-invasive approach that helps modulate the Autonomic Nervous System (ANS) through Resonance Frequency (RF) breathing, supporting CR for cardiovascular patients. However, traditional HRVB techniques rely heavily on manual RF selection and face-to-face guidance, limiting their widespread application, particularly in home-based CR. To address these limitations, we propose a remote human-computer collaborative HRVB system, “FreeResp”, which features autonomous RF adjustment through a simplified cognitive computational model, eliminating the reliance on therapists. Furthermore, the system integrates wearable technology and the Internet of Things (IoT) to support remote monitoring and personalized interventions. By incorporating tactile guidance technology with an airbag, the system assists patients in performing diaphragmatic breathing more effectively. FreeResp demonstrated high consistency with conventional HRVB methods in determining RF values (22/24) from 24 valid training samples. Moreover, a one-month home-based RF breathing training using FreeResp showed significant improvements in Heart Rate Variability (HRV) (*p* < 0.05). These findings suggest that FreeResp is a promising solution for home-based CR, offering timely and precise interventions and providing a new approach to long-term cardiovascular health management.

## 1. Introduction

With the increasing pressures of modern society and shifts in lifestyle [1], cardiovascular diseases have gradually become a global public health challenge [2,3,4,5]. According to the latest statistics from the World Health Organization, the proportion of cardiovascular diseases in the global disease burden continues to rise [6]. Cardiac Rehabilitation (CR), with its treatment strategy based on five core prescriptions, has been widely applied in various cardiovascular diseases. Numerous studies have shown that CR has significant therapeutic benefits for patients with cardiovascular diseases. Not only does it positively impact biological markers of the disease, but it also significantly mitigates postoperative depressive moods and ongoing psychological stress [7,8]. As a result, CR is increasingly perceived as an integral component of continuous care for patients with cardiovascular diseases. Its application is a Class I recommendation in most contemporary cardiovascular clinical practice guidelines [9].

Despite the clinical effectiveness of CR being widely acknowledged, its global application remains limited [10]. Traditional CR programs often require patients to invest a significant amount of time and rely on specific facilities and professionals [11]. In fact, many patients, especially those from low-income and remote areas, find it challenging to access these specialized resources [12]. For most patients, consistently participating in rehabilitation activities and following doctor’s recommendations becomes a significant challenge [13], encompassing issues like time constraints, transportation, and other obstacles. Moreover, fear of exercise exacerbating the disease becomes a primary barrier to participation in rehabilitation [14].

In recent years, Heart Rate Variability Biofeedback (HRVB) has been gradually introduced as a supplementary treatment strategy for CR [15]. As a simple and non-invasive respiratory feedback method, HRVB primarily focuses on modulating the Autonomic Nervous System (ANS) [16], especially the balance between the sympathetic and parasympathetic systems. By adjusting the breathing rate to Resonance Frequency (RF), HRVB can effectively enhance a patient’s Heart Rate Variability (HRV) [17]. Notably, HRV is not only considered an essential indicator of cardiac health but also closely linked with psychological health and overall well-being. Methods for obtaining HRV have become increasingly diverse, encompassing traditional ECG and PPG techniques as well as emerging non-contact and portable approaches. For example, Smart Photonic Wristbands achieve high-precision pulse monitoring through speckle pattern analysis [18], Millimeter-Wave Radar utilizes thoracic micro-movements to enable non-contact HRV acquisition [19], and Ballistocardiography records the mechanical vibrations of cardiac activity using piezoelectric sensors [20]. Additionally, commercial wearable devices, such as the Apple Watch, are increasingly used in health management by integrating intelligent algorithms [21].

The non-invasiveness and ease of implementation of HRVB make it an ideal supplement in the field of CR, assisting patients in overcoming concerns and fears about excessive exercise. Additionally, HRVB can provide real-time feedback, enabling doctors to better understand and adjust patients’ physiological states, thereby strengthening patients’ self-management awareness and treatment participation. In terms of short-term efficacy, just six sessions of HRVB can significantly restore the autonomic function of the heart [22,23]. In terms of long-term effects, research by Yu et al. [24]. has revealed the marked effectiveness of HRVB in improving cardiovascular prognosis and enhancing the heart’s autonomic homeostasis. Importantly, HRVB not only focuses on patients’ physiological recovery but also encompasses psychological rehabilitation, assisting patients in coping with the mental stress resulting from cardiovascular diseases, aligning closely with the core objectives of CR [15].

In current HRVB research, the majority of experimental designs follow the process outlined by Lehrer [17]. Patients need to determine their RF under the guidance of professional therapists (or doctors) and subsequently participate in lab consultations at regular intervals, such as weekly. The laboratory training also includes training in diaphragmatic breathing and pursed-lip breathing techniques to ensure achieving the optimal RF without hyperventilation [25]. Therapists analyze patients’ physiological signal data, optimize RF settings based on this, and evaluate training effects [26]. Outside of consultation periods, patients maintain daily training sessions of around 20 min at home, 4 to 6 days a week, with the entire training cycle typically spanning about four weeks. Visual and auditory guides ensure that patients maintain the RF breathing rate during each training session and engage in abdominal breathing, which is another aspect the therapist must pay attention to.

However, given that CR is often a prolonged process, there are challenges in applying HRVB more broadly in the field of CR. Especially when patients are discharged and rehabilitation training shifts from the clinical setting to daily life and home environments, training and individualized adjustments in HRVB become more challenging. For instance, home environments might be filled with various distractions, such as household responsibilities, work pressures, and environmental noise [27]. The participants might find it hard to maintain attention during training due to fatigue, impacting HRVB’s integrity and accuracy [28]. Particularly in the absence of professional medical supervision, training quality largely depends on patients’ self-discipline. If training is interrupted for any reason, such as fatigue or distraction, the effectiveness of the entire training cycle might be called into question, and doctors often find it challenging to obtain this information promptly. More importantly, doctors need to not only select the RF for patients but also evaluate the training’s effectiveness, adding to their workload.

Therefore, adjusting the HRVB training strategy, integrating real-time data collection and analysis into a human-machine collaborative cognitive decision-making framework, and designing an HRVB support system tailored for CR have profound significance for the further application and promotion of HRVB in the field of CR. This can more swiftly identify potential challenges patients might face during rehabilitation, offering timely intervention measures. Thanks to advancements in cognitive computing, it is now feasible to construct cognitive models to assist therapists in decision-making and workflows [2,4,29,30]. Specifically, two core aspects of HRVB therapy—RF selection and real-time training monitoring—can be enhanced through the integration of cognitive models. For this, we designed and implemented a personalized biofeedback system named “FreeResp”. This system not only combines the advantages of wearable and Internet of Things (IoT) technology [31] but also incorporates a simple human-machine collaborative cognitive decision-making framework. In terms of hardware, we successfully integrated sensors into a wearable garment, allowing for comfortable and accurate data capture, even in a home setting. To enhance the user experience, we also added an inflatable module to guide users in diaphragmatic breathing, making the entire training process more intuitive and immersive. Through IoT technology, real-time data can be uploaded to the cloud [32], enabling remote monitoring and guidance of user training activities by therapists. On the other hand, the system also explored a simplified cognitive model that automatically selects the most suitable RF by analyzing HRV metrics, eliminating the need for direct therapist intervention. This innovative approach not only eliminates the need for manual data uploading by patients but also ensures that therapists can stay informed of the patient’s training status at any moment, thereby guaranteeing the continuity and accuracy of the training.

The rest of this paper is structured as follows: Section 2 delves into our main contributions from a system design perspective and details the experimental design. Section 3 presents the results, Section 4 discusses the findings, and finally, Section 5 concludes the paper.

## 2. Materials and Methods

**Figure 1:**
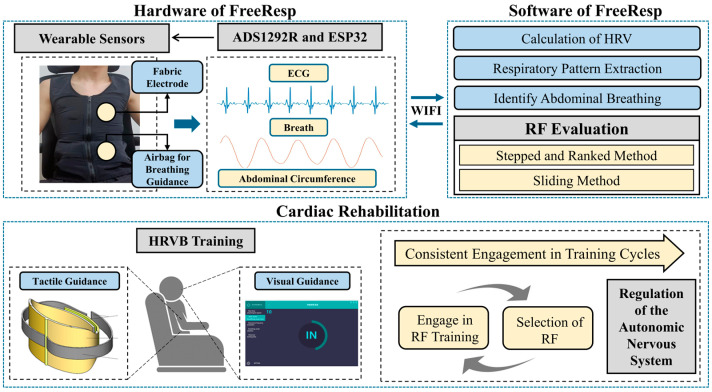
The overall architecture of the system, “FreeResp”.

With the rapid development of IoT technology, continuous and real-time physiological signal tracking and monitoring have become easily accessible [32]. Especially for CR patients [33], continuous physiological monitoring is crucial for their recovery process [34,35,36]. Against this backdrop, we have constructed a simple human-machine collaborative cognitive decision-making system based on wearable devices, as shown in Figure 1. This system not only serves as a bridge, enabling remote therapists to provide timely guidance and intervention based on real-time data but also employs algorithms to automatically assess whether users have reached the RF breathing state. Furthermore, we introduced a method that empowers patients to select the RF independently, which is particularly pivotal in CR, given the paramount importance of the RF breathing state in enhancing cardiac function and promoting recovery [15,26].

### 2.1. Development of Personalized Biofeedback Training Prototype

#### 2.1.1. Development of Physiological Information Detection Device

**Figure 2:**
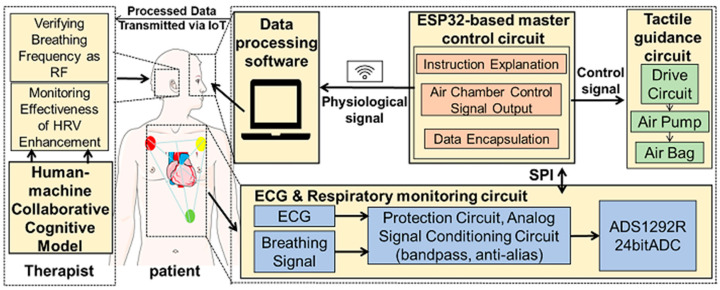
System hardware framework.

Considering the potential application of HRVB in the field of CR, the collection of ECG is essential. Compared to PPG signals, ECG can be better used to identify and assess many cardiac diseases. Studies have shown that using ECG for calculating heart rate is more accurate than using PPG [37], especially when studying psychological conditions such as anxiety disorders [26,38]. We have developed a portable physiological data acquisition circuit capable of measuring ECG and other vital signals. This device is built on the ADS1292R (Texas Instruments, Dallas, TX, USA) chip and ESP32 (Espressif Systems, Shanghai, China) chip. It can monitor diaphragmatic breathing conditions in real time. The specific design is illustrated in Figure 2.

The ADS1292R chip serves as the core acquisition module, capturing subtle changes in the ECG signal with a 24-bit high resolution. The acquired signals are initially processed to eliminate external interference. These signals then pass through a second-order Sallen-Key filter in the analog signal conditioning circuit to remove high-frequency noise, while the anti-aliasing filter function is used to retain key ECG information [39]. The processed signals are transmitted to the ESP32 chip via the SPI interface. The ESP32 chip plays multiple roles in this system, not only receiving and packaging the processed signals but also uploading the data to the cloud for real-time analysis via wireless communication technology. Additionally, the ESP32 chip generates control signals to adjust the operation of the tactile guidance module, ensuring that the user’s breathing pattern remains synchronized with the target frequency.

Furthermore, to improve the quality of the ECG signal and effectively reduce motion artifacts and other sources of noise, a denoising algorithm based on a diffusion model is deployed on the cloud [40]. This algorithm uses a trained diffusion model for reverse denoising and signal reconstruction, effectively removing noise and maximizing the restoration of the signal’s original physiological features, especially the R-wave characteristics. The ECG signals processed by this algorithm provide more accurate and high-quality data support for subsequent HRV analysis.

#### 2.1.2. Development of Daily Training Breathing Assistance Device

During traditional HRVB training, breathing guidance is typically presented to patients in animated form, where graphical expansion and contraction or ascent and descent represent the two phases of exhalation and inhalation. However, focusing excessively on these animations can be fatiguing for patients, especially when they need to keep their eyes open for extended periods to follow the breathing guidance. For patients unfamiliar with diaphragmatic breathing, maintaining it for extended periods can be challenging. These factors can influence RF determination and may weaken training effects. Although therapists can immediately assess and intervene in the user’s training status during the process, they may not be able to correct mistakes in long-term training scenarios in a timely manner.

This paper designs a scheme to guide RF breathing training through tactile feedback. Alongside providing graphics-based visual feedback, we introduce airbags as tactile feedback devices. The airbags inflate and deflate on the user’s abdomen or back in sync with breathing, helping reduce the discrepancy between the actual breathing rate and RF and promptly correcting breathing posture. The elasticity of the airbag also alleviates pressure caused by the user’s breathing rate not being in sync with the guided rate. This method, combining visual and tactile feedback, not only reduces the user’s attention required during training but also offers more intuitive guidance and assists in correcting their diaphragmatic breathing technique.

During the RF breathing training process, when the system detects a continuous decline in the amplitude of the HR curve, it automatically fine-tunes the guiding frequency of the airbag or animation. Specifically, the increment is prioritized at 0.25 BPM, aiming to approach the resting breathing rate. From this baseline frequency, the breathing rate is constantly fine-tuned to ensure the stability of the HR curve amplitude. With the integration of the cognitive decision-making model, the system can in real time determine whether patients have successfully reached the RF breathing state, thus offering a more personalized and intelligent training experience.

#### 2.1.3. Human-Machine Cognitive Decision-Making in HRVB Training

**Figure 3:**
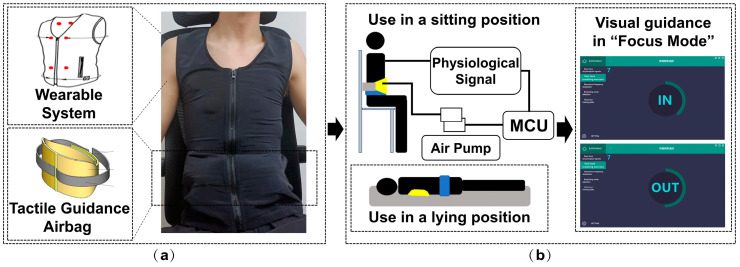
The usage method of FreeResp: (**a**) RF-breathing training system and its (**b**) two tactile guidance positions.

FreeResp features an integrated, wearable design. Electrodes and circuits are crafted from specialized fabric, embedded within the clothing, and connected to the physiological information collection module, eliminating consumable losses and allergy risks associated with conventional ECG devices. The clothing’s abdominal and back areas are designed with ample space to incorporate airbags, allowing for minor adjustments based on user requirements. Figure 3 depicts a user wearing this integrated innerwear for data collection. By simply wearing this specially designed garment, patients can undergo the entire HRVB training process, reducing the hassle of additional operations and devices.

Importantly, our cognitive decision-making model substantially reduces the workload of therapists in monitoring patients. Alerts to therapists are only initiated when the system detects repeated deviations in the user’s breathing depth or frequency. In conjunction with the RF breathing evaluation method, therapists can determine the real-time breathing status of patients. This data are instantly transmitted to therapists who can then immediately provide feedback via social software or phone, intervening in the user’s training. This immediate guidance and recommendation pave the way for a human-machine collaborative cognitive decision-making model in HRVB training.

### 2.2. Cognitive Model for RF Analysis and Selection

In order to integrate HRVB as a supplementary method into the daily and home CR of heart disease patients, long-term, independent, and personalized training becomes crucial [41]. However, various factors in remote home environments, such as life stress or certain illnesses, can influence a user’s physiological state. Traditional methods for determining RF have often been limited by specific laboratory conditions and short-term observations, relying on the expert judgment of therapists. Currently, many studies only conduct a single RF assessment at the outset, with some defaulting to a respiratory rate of 6 BPM [26]. Notably, the stability and adaptability of RF throughout continued training remain undefined [42]. Thus, ensuring that patients consistently adopt the optimal breathing rate during daily training makes repeated RF evaluations especially critical. This study introduces a therapist-independent method for selecting RF, notable for its ability to adapt to the immediate physiological needs of the patient and reduce the time and financial burdens of frequent expert consultations.

**Figure 4:**
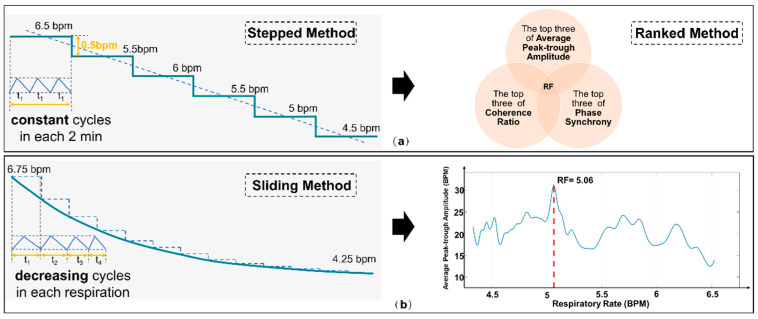
Schematic diagram comparing (**a**) the “Stepped and Ranked Method” and (**b**) the “Sliding Method”.

Historically, Lehrer’s “Stepped Method” was employed to determine the individualized RF that maximizes stress reflex. Recently, Lehrer introduced the “Sliding Method”, which fine-tunes the length of each breathing cycle [43], aiming to pinpoint the appropriate breathing rate in a shorter timeframe. Subsequent comparisons of HRV within a fixed time window, coupled with RF assessments, allow for deducing the respiratory RF. As illustrated in Figure 4, our developed system integrates both strategies, giving patients the choice between the “Stepped Method” and the “Sliding Method”.

However, traditional methods require the assistance of therapists and the processing of physiological information, tasks that patients cannot complete independently. To address this issue, we introduced the “Stepped and Ranked Method” based on the cognitive decision-making model as a therapist-independent RF selection technique. This method integrates the expertise of human professionals with the computational power of machines, aiming to quickly adapt to patients’ immediate physiological needs, thereby reducing the necessity for frequent consultations with experts. This method aims to provide a reliable and scientific approach for identifying the best matching breathing frequency with resonant effects in an environment without the supervision of a professional therapist. Traditionally, following existing guidelines, therapists rely on six standard metrics to evaluate the effectiveness of each breathing frequency. However, to achieve this objective in a home setting, we have adopted an integrated approach using three key HRV metrics: Average Peak-to-Trough Amplitude, Phase Synchrony, and Coherence Ratio [38].

These three metrics undergo cross-validation and are used to holistically assess the effectiveness of various breathing frequencies. Specifically, we rank the breathing frequency values corresponding to these three metrics in descending order and then select the top three from each metric for further analysis. If a particular frequency appears in the top three across all three metrics, it is confirmed as the optimal RF. In instances where multiple frequencies meet this criterion, the frequency with the largest average peak-to-trough amplitude is chosen as the RF.

Through this comprehensive assessment strategy, we are able to scientifically and accurately identify the breathing frequency most closely related to resonant effects, even in the absence of a therapist’s involvement. As illustrated in Figure 4a, this method offers us a more precise and reliable means for the quantitative evaluation of RF.

### 2.3. Experimental Design

We designed an experiment primarily to evaluate the application effectiveness and suitability of the system in remote and home environments. The benefits of HRVB in the CR field have been substantiated by numerous academic studies. Therefore, to further its broader application in the field of CR, especially for the rehabilitation of heart disease patients in long-term home settings, our experiment shifted its focus to verifying the system’s suitability and effectiveness, rather than merely demonstrating the effects of HRVB. In light of this, we selected university students as our test subjects because this group is more readily accessible. Throughout the entire experimental process, we used the HRV indicators of these university students as the core evaluation criteria for assessing the application results of our system.

This study was approved by the Scientific Research Ethics Committee of the General Hospital of Southern Theatre Command (approval number: NZLLKZ2022154). All participants provided written informed consent prior to the experiment. All experimental procedures strictly adhered to the ethical principles outlined in the Declaration of Helsinki (revised in 2013). Additionally, all data collected during the study were anonymized to ensure the privacy of the participants.

The first experiment aimed to validate the accuracy of the fabric electrodes used by FreeResp in acquiring HRV data. Ten healthy participants were recruited, with Ag/AgCl electrodes serving as the standard. During the experiment, the placement of both Ag/AgCl and fabric electrodes was carefully aligned, with both positioned below the midclavicular line to ensure consistency in signal origin. Single-lead ECG signals from both electrode types were synchronously recorded using the g.Hlamp device (manufactured by g.tec). The data collection was conducted under two conditions: resting and controlled breathing.

In the resting condition, each participant rested for 5 min while their ECG data were recorded. In the controlled breathing condition, participants performed breathing at a frequency of 6 BPM for 5 min. The experimental data were used to evaluate the performance of the two electrode types in terms of HRV time-domain metrics (e.g., SDNN, RMSSD) and frequency-domain metrics (e.g., LF, HF power). Finally, consistency analysis methods, such as Bland-Altman plots, were used to compare and evaluate the measurement consistency of the two electrode types under different conditions.

Upon completion of the electrode performance validation, the experiment proceeded to the system’s application and validation phase. This phase focused on assessing the effectiveness and applicability of the system in remote and home-based environments.

**Figure 5:**
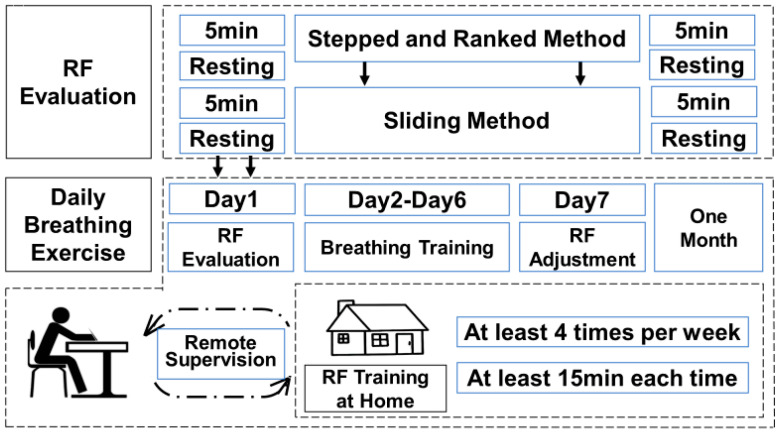
Schematic diagram of the experimental procedure.

In the second phase of the experiment, which focused on the application and validation of the system, we recruited 30 male university students aged between 18 and 24 years as participants. The overall experimental setup is illustrated in Figure 5. All participants were healthy individuals who had not taken any psychotropic drugs, asthma medications, or cardiac drugs, nor had they undergone any psychological therapy prior to the experiment. Additionally, participants were instructed to avoid smoking, drinking alcohol, or engaging in strenuous exercise for at least one hour before the experiment. Before the start of the experiment, the height, weight, and BMI of all participants were measured.

All participants were first familiarized with the concepts and techniques of RF breathing in a laboratory environment. The research team demonstrated the correct technique for diaphragmatic breathing and guided participants in performing slow breathing at a frequency of 6 BPM. During the training, the research team observed participants’ blink frequency to assess their attention and fatigue levels. Next, participants underwent training using the “Stepped and Ranked Method”, which involved decreasing the breathing rate from 8 BPM to 4.5 BPM in increments of 0.5 BPM, with each frequency maintained for 3 min. Upon completing this training, 10 participants were randomly selected to undergo assessment using the “Sliding Method”.

Subsequently, one participant was selected to engage in daily RF breathing training for a period of four weeks. During this phase, the participant performed RF breathing training daily, with the training progress adjusted in real-time by the therapist using the human-machine collaborative decision-making model. The therapist monitored the participant’s HRV changes to determine whether adjustments to the training schedule were needed. If the participant experienced fatigue, fell asleep, or encountered other issues during the training session, the therapist intervened promptly and restarted the training to ensure its effectiveness.

## 3. Results

### 3.1. Signal Quality Testing of Wearable Devices

**Figure 6:**
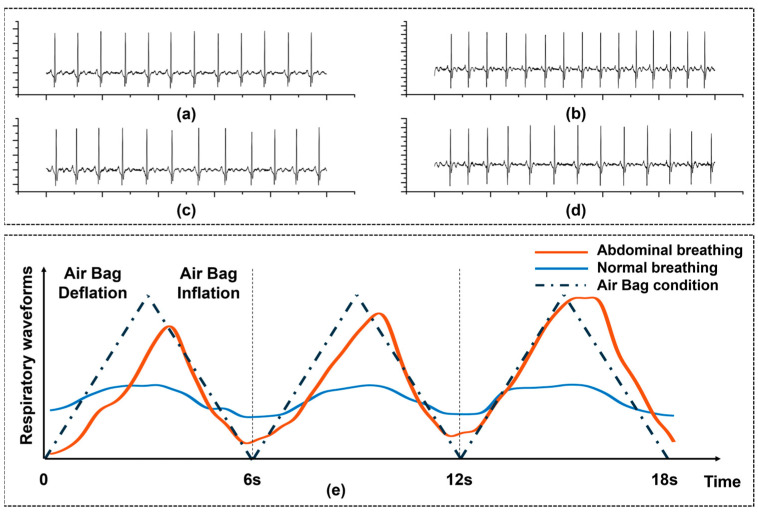
The impact of ’FreeResp’ on physiological signals. (**a**) Resting single-lead ECG was collected using standard Ag-AgCl electrodes. (**b**) Resting single-lead ECG was collected using textile electrodes at the same location. (**c**) ECG was collected under slow breathing conditions using standard Ag-AgCl electrodes. (**d**) ECG was collected under slow breathing conditions using textile electrodes. Each ECG was collected for a duration of 10 s. (**e**) Comparison of changes in abdominal circumference during breathing, with and without the guidance of the airbag.

Prior to conducting the overall RF experiment, we first validated the performance and feasibility of our designed wearable shirt for ECG data collection. We compared the performance of our ECG shirt with conventional ECG electrodes during RF-breathing training, as shown in Figure 6a. Our ECG shirt demonstrated no significant difference in ECG signal acquisition compared to conventional ECG electrodes during both resting and low-frequency breathing states. The R-wave feature in the ECG signal was clear, indicating that we could obtain the required HRV-related indicators.

**Figure 7:**
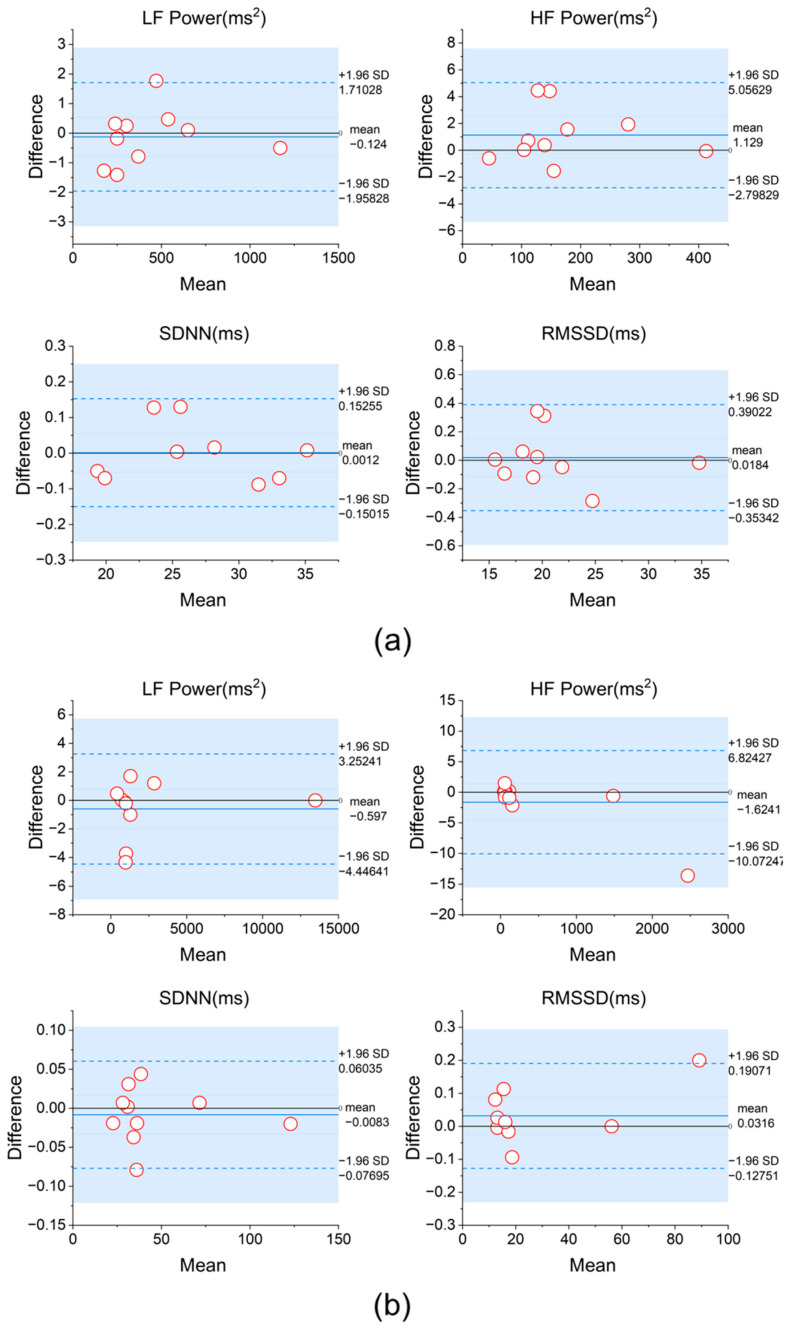
(**a**) Bland-Altman analysis of HRV metrics obtained using two types of electrodes during the resting state and (**b**) during 6 BPM breathing.

To further validate the consistency between fabric electrodes and traditional Ag/AgCl electrodes in HRV measurements, we conducted a Bland-Altman analysis to compare the data collected by the two electrode types under resting and low-frequency breathing conditions. As shown in Figure 7a,b, the differences between the two electrode types were minimal in terms of HRV time-domain metrics (e.g., SDNN, RMSSD) and frequency-domain metrics (e.g., LF, HF power), with the vast majority of data points falling within the 95% consistency limits.

Additionally, we tested the effect of the airbag in guiding subjects’ diaphragmatic breathing. Figure 6e shows the comparison of abdominal circumference between a subject’s breathing without the airbag and their breathing guided by the airbag. The airbag is designed to assist users who are unable to maintain diaphragmatic breathing for extended periods, helping them maintain a stable breathing pattern. For some participants, under conditions without the airbag, although the abdominal circumference changes were initially maintained within a certain range, as the number of breaths increased, distractions or abdominal fatigue caused the range of abdominal circumference changes to gradually narrow, while the breathing rhythm fluctuated or degraded into chest breathing. In contrast, under airbag guidance, tactile feedback effectively enhanced the subject’s active abdominal effort by providing real-time physical cues, making the range of abdominal circumference changes more stable, and significantly optimizing the breathing rhythm.

### 3.2. Stepped and Ranked Method

We successfully recruited 30 healthy adult male participants for respiratory training. Upon the completion of HRVB, one participant withdrew from the study due to difficulties in mastering diaphragmatic breathing. The remaining participants successfully completed the slow-breathing experience with a target of 6 BPM. However, during the process of RF assessment, five participants were unable to continue due to fatigue and attentional deficits; one among them requested to temporarily halt the experiment for rest. In remote CR training, especially during the long-term training process, the requirement for sustained attention in daily HRVB training becomes considerably challenging in the absence of on-site supervision by a therapist, particularly over extended training periods [44,45]. The data from these six participants were excluded, and further analysis and processing were conducted on the remaining 24 participants. This involved selecting the high-ranking (top three) frequencies of three indicators: “HRmax-HRmin (Peak-trough amplitude)”, “percent total LF power as LF/(LF + HF)”, and “Phase synchrony”. Table 1 shows the ranking data of one participant’s experiment [38].

Therefore, this participant’s top three rankings for HRmax-HRmin were 4.5/5/5.5, for LF power were 4.5/5/6, and for phase synchrony were 4.5/5/6.5. The participant’s RF was 4.5 BPM. It is important to note that the RF doesn’t necessarily rank first across all indicators; it might only rank first in two or even just one of the indicators.

**Figure 8:**
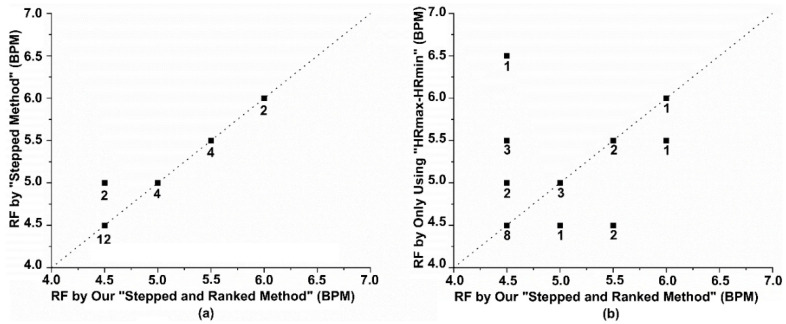
Analysis of the effectiveness of our method in determining RF (**a**) A comparison of the RF determined by our method and the manually judged RF. (**b**) A comparison of the RF determined by our method and the RF determined using only time-domain indicators. The numbers on the dots represent the number of participants.

We first compared the RF results of all participants with the RF values determined by evaluators through guidelines. Before the evaluators determined the RF values of the participants, they were unaware of the values calculated by the new weighting method. The RF values of the 24 participants are shown in Figure 8a, with 22 participants having consistent RF values. For the participants with inconsistent results, 4.5 BPM and 5 BPM were both in the top two rankings for all three evaluation indicators. The manually determined method selected the highest-ranking indicator for HRmax-HRmin, while our method selected 4.5 BPM, which ranked first in the other two indicators. Although the results differed, the difference was only 0.5 BPM, and the effect on training still needs to be compared through long-term training. In addition, to improve the efficiency of determining the RF, we attempted to abandon frequency domain indicators and used only time domain indicators (HRmax-HRmin ranking) as the basis for RF determination. The determined RF is shown in Figure 8b.

### 3.3. Differences Between the Different Methods

In this study, we conducted a comprehensive comparative analysis between the “Stepped and Ranked Method” and the “Sliding Method”. Our primary objective was to rigorously evaluate the accuracy and reliability of both approaches in determining RF while also exploring a strategy that could combine the advantages of both methods, facilitating the determination of RF for patients even in remote settings without guidance.

We selected 10 willing participants, and their physiological information is provided in Table 2. We then obtained their RF values using the sliding method implemented in FreeResp. These values were then compared with those determined through the “Stepped and Ranked Method”. It is noteworthy that the sliding method predominantly relies on time-domain metrics for evaluation. The comparative results of both methodologies have been detailed in Table 3.

**Figure 9:**
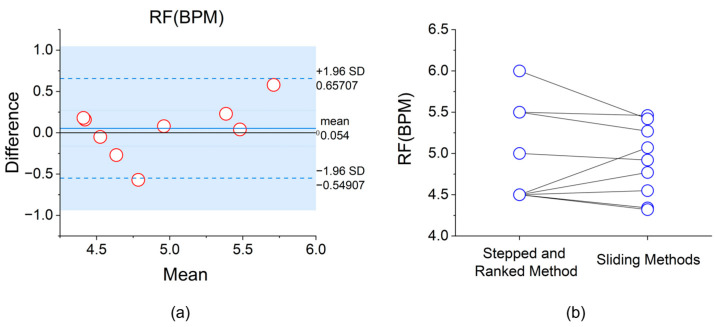
(**a**) Bland–Altman analysis plot of RF determination based on the “stepped and ranked method” and the “sliding method”; (**b**) Before-and-after comparison graph of RF determination based on the two methods.

Experimental results were analyzed using IBM SPSS Statistics 27. Among the 10 participants, 8 showed a difference in RF values between the two methods within 0.5 BPM, demonstrating high consistency. The remaining 2 participants had differences of less than 0.6 BPM, specifically −0.57 BPM (P6) and 0.58 BPM (P7). Further analysis revealed that in the sliding method, the RF values of 3 participants were higher than those obtained using the stepped and ranked method (P5, P6, P8), with an average decrease of 0.30 BPM. The RF values of the remaining 7 participants decreased (P1, P2, P3, P4, P7, P9, P10), with an average increase of 0.20 BPM. The Bland–Altman plot shown in Figure 9a indicates that the mean difference between the two methods was 0.054 BPM, with the 95% consistency limits ranging from [−0.549, 0.657] BPM, suggesting a high overall consistency between the methods. Additionally, the before-and-after comparison graph more intuitively demonstrates the direction and magnitude of the RF value changes after using the sliding method. Using the Wilcoxon signed-rank test, there was no significant difference between the “sliding method” RF values and our method values (*p* > 0.05).

### 3.4. Breathing Training in Home Settings

This study employed HRV metrics to investigate whether participants could effectively regulate the balance of their ANS during each training session under the supervision of a human-machine collaborative cognitive decision-making model. We randomly selected one volunteer and provided a detailed explanation of the daily training guidelines. Over the following month, the participant engaged in continuous daily training within the comfort of their home environment, without the need to return to the laboratory.

During the experiment, researchers monitored the participants’ physiological signals in real-time based on the human-machine collaborative cognitive decision-making model to assess the effectiveness of the training. If it was discovered that a participant had forgotten to train for some reason, or if the training outcomes were not satisfactory, researchers would immediately intervene and communicate with the participant to adjust the training strategy. We required each participant to train at least four times per week, with each session lasting at least 15 min. Furthermore, participants were asked to re-evaluate their RF every week using the “stepped and ranked method”. Ultimately, we successfully collected complete training data for one participant over a 27-day period. Among these 27 training sessions, the participant forgot to train five times due to personal reasons but made up for those sessions following reminders from the researchers.

**Figure 10:**
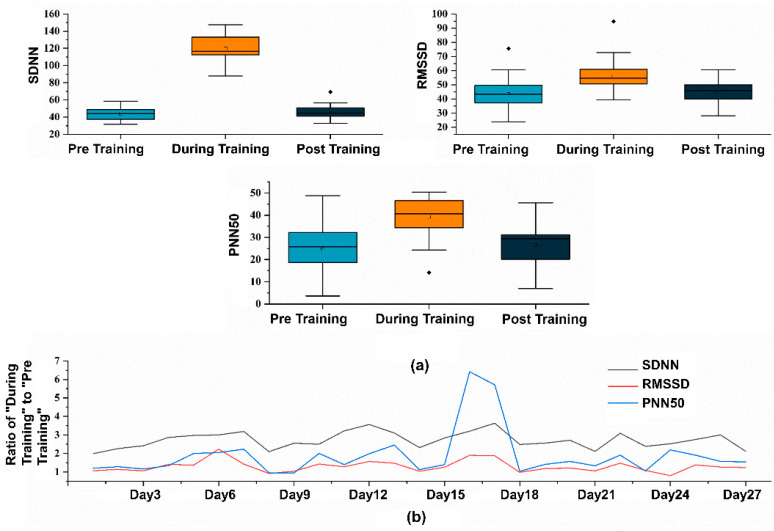
(**a**) Statistical graphs of various HRV metrics before, during, and after the training sessions. (**b**) Ratios of “During Training” to “Pre Training” across 27 training sessions.

We chose HRV as the indicator to assess the effectiveness of remote CR [46], which includes the Standard Deviation of RR Intervals (SDNN), the Root Mean Square of Successive Differences (RMSSD) between adjacent heartbeats, and the Proportion of RR Intervals Greater than 50 Milliseconds (PNN50). An improvement in these metrics signifies the effectiveness and reliability of the training data [47]. SDNN measures the standard deviation of R-peak intervals, with larger SDNN values indicating higher HRV. RMSSD measures the difference between adjacent HR and serves as a time domain measure of HRV. The data results, as shown in Figure 10a, indicate that unsupervised RF-breathing training using FreeResp effectively improves various HRV indices (*p* < 0.05). Figure 10b shows the ratio of HRV data during training to pre-training HRV data, which can reflect the activation state of the participant during the training process.

## 4. Discussion

In this study, we first delineate the essential features and improvements that an HRVB system tailored for CR should embody. Specifically, these are:1. *Longevity and Sustainability:* Ensuring that the system can accompany patients for long-term use. 2. *Adaptability:* Adjusting training plans based on the unique needs and progress of each patient. 3. *Real-time Monitoring and Feedback:* Making training more accurate while providing crucial data to medical professionals. 4. *User-Friendliness and Portability:* Guaranteeing a system that is straightforward and adaptable to various environments.

To address these core elements, our research employed fabric electrodes, designed the ’Stepped and Ranked Method’ model, harnessed IoT technology, and innovatively crafted tactile guidance airbags.

The wearable shirt we designed incorporates fabric electrode technology, which, compared to traditional ECG electrodes, offers greater wearing comfort and convenience [48], reducing discomfort and allergy risks associated with prolonged use. [49]. The experimental results show that the ECG signal acquisition performance of the fabric electrodes under resting and low-frequency breathing conditions is highly consistent with traditional electrodes. In the RF selection process, since we focus more on the relative ranking of HRV metrics across different breathing frequencies rather than their absolute values, the slight deviation of the fabric electrodes has minimal impact on RF determination.

Chest breathing is the most common breathing method; however, under this method, the diaphragm is not effectively activated, leading to relatively small changes in abdominal circumference and low tidal volume, making it difficult to achieve the desired training effect [26,38]. In this study, the use of an airbag guide effectively helped participants achieve diaphragmatic breathing, significantly increasing abdominal circumference changes and reflecting an improvement in breathing depth. Larger abdominal circumference changes not only indicate successful activation of the diaphragm [41] but may also further enhance breathing efficiency and oxygen intake, which is crucial for patients undergoing CR [50].

Overall, our results show that our cognitive decision-making model can effectively determine the RF-breathing frequency value in a non-supervised setting, avoiding the limitations of single-indicator evaluations. Participants have the capability to autonomously determine their RF within the confines of their home environment, obviating the need for frequent laboratory visits. This saves the patient’s time and greatly reduces the therapist’s workload, allowing clinicians to focus more intently on tailoring daily training regimens to ensure the efficacy of each session. Additionally, within the setting of home-based self-management, patients are afforded the flexibility to adjust their breathing frequencies, thereby becoming familiar with and mastering RF breathing techniques. This not only allows for more extended, personalized, and confidential training sessions under remote supervision but also promotes a deeper integration for CR, providing more personalized treatment options for patients.

This study compared the performance of the “Stepped and Ranked Method” and the “Sliding Method” in determining RF. The results showed that both methods exhibited high consistency, with the majority of participants showing differences within 0.5 BPM. The Bland-Altman analysis indicated that the differences between the two methods were mainly within the 95% consistency limits, validating their reliability. Nevertheless, the results of the “Sliding Method” showed some fluctuation in certain participants, which may be related to its reliance primarily on time-domain metrics, while the “Stepped Method”, which integrates multiple metrics, exhibited more stable performance. Although no significant statistical difference was found between the two methods, individual differences may still have a potential impact on the long-term effectiveness of HRVB training, requiring further research for validation [43]. Despite the high consistency, the “Sliding Method” cannot fully replace the “Stepped Method”. The latter, although time-consuming and complex, offers a comprehensive assessment from both time-domain and frequency-domain metrics. On the other hand, the “Sliding Method” has advantages in time efficiency and operational simplicity, and it provides more granular RF values. However, whether this granularity can significantly enhance HRVB effectiveness still needs verification. Currently, visual cues remain the primary method for guiding patients’ breathing frequency, but small differences may be difficult for patients to perceive. Moreover, while the “Stepped Method” requires three minutes of breathing at each frequency, the “Sliding Method” only requires one breath per frequency, which may result in weaker HRV changes for some individuals.

Therefore, our method serves as a strategic approach aimed at comprehensively evaluating a broad frequency spectrum, thereby providing a holistic profile of RF. In contrast, the “Sliding Method” excels in operational ease and granularity. Capitalizing on the strengths of both methods, we propose a new hybrid strategy for RF determination. Specifically, an initial RF value is first identified using the “Stepped and Ranked Method”, which then serves as the benchmark for a ± 0.5 BPM range for further fine-tuning using the “Sliding Method”. For individuals who engage in long-term self-management at home, it is recommended to use our method on a monthly basis to gauge the RF range and then fine-tune it before each training session or weekly using the “Sliding Method”, aiming for more personalized training. In the future, we plan to expand the sample size to further investigate the impact of fine-tuning RF values to two decimal places on training outcomes.

RF breathing has already been preliminarily validated in various health management areas, including hypertension, anxiety disorders, and insomnia. The FreeResp system, through optimized hardware design and data acquisition capabilities, can further expand its applicability. For example, in everyday environments, the system will integrate more HRV signal acquisition methods and enhance its portability and applicability through a lightweight device design. In the fields of sports rehabilitation and performance optimization, FreeResp can ensure stable training signals in exercise conditions by improving motion artifact removal, thereby providing athletes with reliable HRV monitoring and training feedback. Moreover, the IoT functionality of the FreeResp system offers more possibilities for community-based rehabilitation. Through remote monitoring and data analysis, therapists can efficiently manage rehabilitation plans for multiple patients and generate personalized training reports. This not only improves the utilization efficiency of rehabilitation resources but also lays the foundation for future widespread applications in community settings. However, the realization of community rehabilitation requires higher standards for hardware cost optimization, data transmission stability, and security. Therefore, these areas will be the focus of future improvements to ensure that FreeResp can cover a broader population and use scenarios.

The limitations of this experimental study include a short duration and a small number of participants. Healthy young adult participants underwent breathing training for only one month, which may be why there were no significant differences in their HRV indices before and after training. It is expected that with long-term continuous training, participants will gradually develop the habit of RF-breathing training, which will help improve patients’ physiological adaptability.

## 5. Conclusions

This study introduces FreeResp, an HRVB training system specifically designed for CR patients. FreeResp, by integrating wearable technology and IoT functionality, not only enables real-time collection and analysis of patients’ physiological signals but also provides personalized RF adjustments through a human-computer collaborative cognitive decision-making model. First, compared to traditional ECG electrodes, the fabric electrodes used in FreeResp significantly improve wearing comfort while avoiding the hassle of frequent electrode replacement, allowing patients to continue long-duration HRVB training in a home environment. Additionally, the system’s airbag guidance technology is one of its key features, effectively promoting diaphragmatic breathing and helping patients perform deep breathing, thereby improving autonomic nervous system function. This feature is especially important for patients who struggle to maintain diaphragmatic breathing. Another highlight of FreeResp is its ability to autonomously adjust RF based on real-time physiological data. Through the human-computer collaborative cognitive decision-making model, the system can automatically select RF without the need for therapist intervention, thereby reducing the workload of therapists and enabling remote supervision. Furthermore, the system utilizes IoT technology to facilitate remote data monitoring and real-time feedback, ensuring that therapists can always be informed of the patient’s training status, further optimizing treatment outcomes. In summary, FreeResp provides an efficient, comfortable, and personalized HRVB training platform for cardiac rehabilitation patients and offers convenient remote monitoring and intervention tools for home-based rehabilitation, demonstrating significant practical value.

Despite the encouraging results, the study does have some limitations, such as a relatively small sample size that did not comprise cardiac patients and the absence of an appropriate control group. Future research endeavors will focus on expanding the sample size and conducting more diversified experimental validations. Additionally, given that motion artifact removal still presents challenges in dynamic home environments, we plan to build on the existing FreeResp system architecture by incorporating deep learning techniques to collect specific motion artifact data and create a database. This will help optimize and generalize cloud-based noise reduction models. At the same time, we will transfer some artifact processing tasks to edge devices through lightweight design to enhance the algorithm’s real-time processing capabilities and efficiency in complex environments.

## 6. Patents

A granted patent resulting from this work: “Resonance Frequency Breathing Measurement Method, Interactive Prompt Generation Method, Device, and Equipment” (Publication No. CN115517632A).

## Figures and Tables

**Table 1:** Ranking of three HRV indicators for one participant.

Phase Synchrony	HRmax-HRmin	LF Power	RF
4.5/5/6.5/6/5.5	4.5/5/5.5/6/6.5	4.5/5/6/6.5/5.5	4.5

**Table 2:** Demographic and anthropometric characteristics of participants.

Variables	Mean ± SD	Range (Min–Max)
Age (years)	25.6 ± 3.1	22–30
Height (cm)	174.3 ± 5.2	168–183
Weight (kg)	68.5 ± 6.7	60–78
BMI (kg/m^2^)	22.4 ± 1.8	20.0–25.6

**Table 3:** Comparison of the results of the two methods.

Participant	Sliding Methods (BPM)	Our Methods (BPM)	Absolute Difference (BPM)
P 1	4.34	4.5	0.16
P 2	4.92	5	0.08
P 3	5.27	5.5	0.23
P 4	5.46	5.5	0.04
P 5	4.77	4.5	−0.27
P 6	5.07	4.5	−0.57
P 7	5.42	6	0.58
P 8	4.55	4.5	−0.05
P 9	4.34	4.5	0.16
P 10	4.32	4.5	0.18
Mean (absolute value)	4.846	4.900	0.232
SD (absolute value)	0.427	0.538	0.185

## Data Availability

Data will be made available on request.
